# Digital phenotyping in psychiatry

**DOI:** 10.1192/j.eurpsy.2021.71

**Published:** 2021-08-13

**Authors:** L. Orsolini

**Affiliations:** Department Of Clinical Neurosciences/dimsc, Unit of Clinical Psychiatry, School of Medicine, Polytechnic University of Marche, Ancona, Italy

**Keywords:** digital diagnosis, digital psychiatry, digital phenotping

## Abstract

Digital phenotyping represents a new approach aimed at measuring the human behavior by using smartphones and personal device sensors, smartphone apps, keyboard interaction, and various features of subject’s voice and speech. Data collected by a digital phenotyping smartphone application are divided into two categories: a) active data (i.e., those usually collected by using a survey modality) which require an ‘active participation’ from the subject to be generated; and, b) passive data (for instance, those data collected by using Global Positioning System (GPS) traces), usually collected without any participation or action from the subject. Digital phenotyping may theoretically enhance clinicians’ ability to early identify, diagnose and manage any mental health conditions and favoured a more personalized diagnostic and therapeutic approach to several mental conditions. The innovative and insightful approach applied by the digital phenotyping appears to find an interesting and useful application in the field of psychiatry. The digital phenotyping is in line with the new paradigm of the precision psychiatry, i.e. the new approach performed to help clinicians in customizing a psychiatric treatment for each patient, by integrating information about individual phenotypes and genotypes with biographical, clinical and biological data. A precision psychiatry approach would ideally allow clinicians to tailor clinical decision-making and stratify patients to each available treatment according to each one’s likelihood of treatment response and prognosis. Our aims are at providing a comprehensive panorama on evidence-based applications of digital phenotyping in psychiatry.

**Disclosure:**

No significant relationships.

